# Trait-based plant ecology a flawed tool in climate studies? The leaf traits of wild olive that pattern with climate are not those routinely measured

**DOI:** 10.1371/journal.pone.0219908

**Published:** 2019-07-17

**Authors:** Jalal Kassout, Jean-Frederic Terral, John G. Hodgson, Mohammed Ater

**Affiliations:** 1 Equipe bio-Agrodiversité, Laboratoire Botanique Appliquée, Faculté des Sciences, Université Abdelmalek Essaâdi, Tétouan, Morocco; 2 Associated International Laboratory EVOLEA, INEE-CNRS- CNRST, Montpellier, France; 3 Institut des Sciences de l’Evolution, CNRS, IRD, EPHE, Equipe Dynamique de la Biodiversité, Anthropo-Ecologie, Université de Montpellier, Montpellier, France; 4 Unit of Comparative Plant Ecology, University of Sheffield, Sheffield, United Kingdom; 5 School of Archaeology, University of Oxford, Oxford, United Kingdom; University of Poonch Rawalakot, PAKISTAN

## Abstract

Climate-related studies have generally focussed upon physiologically well-defined ‘mechanistic’ traits rather than ‘functional’ ones relating indirectly to resource capture. Nevertheless, field responses to climate are likely to typically include both ‘mechanistic’ specialization to climatic extremes and ‘functional’ strategies that optimize resource acquisition during less climatically-severe periods. Here, this hypothesis was tested. Seventeen traits (six ‘functional’, six ‘mechanistic’ and five ‘intermediate’) were measured from 19 populations of oleaster (wild olive) along a climatic gradient in Morocco. Principal components analysis of the trait dataset identified size and the ‘worldwide leaf economics spectrum’ as PCA axes 1 and 2. However, contrary to our prediction, these axes, and commonly-measured ‘functional’ traits, were little correlated with climate. Instead, PCA 3, perhaps relating to water-use and succulence, together stomatal density, specific leaf water content and leaf shape, patterned with altitude, aridity, rainfall and temperature. We concluded that, at least for slow-growing species, such as oleaster, ‘mechanistic’ traits are key to identifying mechanisms of climatic restriction. Meaningful collaboration between ‘mechanistic’ and ‘functional’ disciplines provides the best way of improving our understanding of the global impacts of climate change on species distribution and performance.

## Introduction

There is a long tradition of studying characteristics of the plant phenotype (traits) that determine how plants respond to environmental factors [1,2]. The measurement of functional (adaptive) traits along environmental gradients has led to the identification of recurrent syndromes of co-occurring plant functional traits or ‘plant functional types’ particularly in relation to resource capture and allocation. These ‘plant functional types’, are a consequence of trade-offs, where traits that facilitate the exploitation of one environment simultaneously reduce fitness in another and, for many, their functional significance is underpinned by ecological theory. In this context, arguably the most fundamental and over-arching set of ecological rules relating plant performance to the environment is provided by CSR strategy theory [3,4]. CSR strategy theory defines two groups of environmental factors that vitally impact upon plant performance. The first, stress (S), includes factors that restrict plant production, particularly mineral nutrients. Other stress factors include suboptimal temperatures and a shortage of light or water. The second group, disturbance (R), results in the destruction of already-produced plant biomass, promoting ruderal growth. Disturbance may result both from the impacts of land use and from extreme climatic events. Where, stress and disturbance are both low, the distribution of species is determined by a third factor, between-plant competition (C). Nevertheless, to date, the use of functional traits has concentrated upon just two areas within strategy theory. The first is the ‘worldwide leaf economics spectrum’, a major factor within stress sensu Grime [3]. This separates species of fertile habitats from those of unproductive ones [5,6]. It is defined both within the classical definition of relative growth rate by Evans [7] and by a fundamental trade-off in leaves between the rapid acquisition of nutrients and the conservation of resources within well-protected tissues [8–11]. The second much-studied topic within strategy theory is plant size. The expression of size is a complex function of the qualitative and temporal opportunities for growth controlled by environmental factors and by interactions with other plants (i.e. combinations of competition, disturbance and stress; Grime [1]). As a consequence of this choice of traits, global meta-analysis within mainstream trait-based plant ecology [12,13] simply identify the ‘worldwide leaf economics spectrum’ and plant size as the two key axes of specialization. Nevertheless, studies of this type, often called ‘trait-based plant ecology’, have been advanced as a key discipline for understanding and advising upon global processes of ecosystem change [6]. A great strength of trait-based plant ecology is that the attributes routinely targeted can be both of ecological significance and easily measured. Numerous species, or even whole floras can be easily categorized with respect to many contrasted ecological dimensions. Moreover, the approach has already been used to compile large and varied trait databases [14]. Encouragingly for the future, many additional traits are available for study [2] and their use may further broaden the scope and utility of trait-based plant ecology.

### Intraspecific variation in trait expression–small changes with large impacts?

To date trait-based plant ecology has primarily focused upon interspecific comparisons [5,15]. In contrast, intraspecific traits, while often equally ecologically significant, have remained comparatively neglected [16–19]. This is unfortunate. As in interspecific comparisons, intraspecific traits frequently pattern instructively along environmental gradients [20,21]. Moreover, intraspecific differences may equal or even exceed those from interspecific studies [22,23]. They are also potentially relevant to climate studies (e.g. tolerance of water stress [24]) as well as, to more general investigations of species coexistence and distribution [25]. These similarities with studies of interspecific traits do not, however, by themselves make a strong case for more intraspecific investigations. However, additionally, and importantly, not all species have the same ecological impact. Amongst, those of greatest ecological significance are canopy dominants, sometimes called ‘ecosystem engineers’ [26,27]. These species monopolize primary resources and, as a result, have a major impact on the ecological functioning of the whole community [26,27]. In order to accommodate these and similar species, we, like Fajardo and Piper [21], Bolnick et al. [17], Albert et al. [28], Laforest-Lapointe et al. [29], Moran et al. [30], Shipley et al. [31], believe that change is needed. An intraspecific dimension should be routinely factored into global models.

### Studying climate–a methodological dilemma

Despite its long-appreciated importance [32], our capacity to predict the potential impacts of climate change upon the world’s flora remains constrained by problems of ecological complexity [33–35]. Moreover, within the context of climate studies the 'functional trait’ approach described above has so far failed to identify and quantify mechanisms of climatic restriction. The most highly-cited paper [36] simply asks the important but preliminary question ‘Which is the better predictor of plant traits, temperature or precipitation?’ More recently, global correlations between traits and climatic variables have also been used to predict possible impacts of climate change on biomass yield [37]. In contrast, an alternative overtly ecophysiological approach has greatly increased our understanding of the impacts of climate on plant performance and survival. Through experimentation, syndromes of ‘mechanistic’ traits conferring tolerance of defined climatic factors have been identified with their function directly interpreted in terms of physics and chemistry. For example, thermal response time of the leaf to changes in surface energy fluxes has been recognized as a major component of physiological tolerance to drought [38,39]. Thermal response time is critical to leaf carbon economics and is defined by leaf traits such as dry matter mass, water mass, specific heat capacity, surface area, width, shape and stomatal density [39]. Similarly, the characteristics of the plant’s hydraulic system additionally regulate response to climate [40,41]. Here, key traits include leaf water potential at turgor-loss point, plant hydraulic conductance or xylem vulnerability [42–44] and, more generally, root architecture [45,46]. Furthermore, encouragingly, Brodribb [47] outlines how simple ‘mechanistic’ traits identified in ecophysiological studies define hydraulic physiology and may be used to explain and predict climatic restriction due to drought.

The contrast between the relatively climatically ineffectual ‘functional’ traits and the more ecophysiologically insightful ‘mechanistic’ traits has catalysed a debate as to how best to advance our understanding of plant-climate interactions [47]. Provocatively, Brodribb writes: ‘By ‘mechanistic’ traits, I mean traits whose function can be clearly physiologically defined, as opposed to the more abstract ‘functional’ traits, such as leaf mass per area (LMA, also known as specific leaf area (SLA)), that have been used to great effect in explaining plant economics over the last 15 years [5]’. Brodribb’s subdivision has been re-enforced by Volaire [48]. Volaire separates ‘mechanistic’ traits as physiological strategies relating to one dominant environmental factor and studied explicitly over short timescales from ‘functional’ traits with ecological strategies relating to multi- environmental factors and studied implicitly over long timescales. However, this debate ignores a key point. ‘Mechanistic’ studies are time-consuming and most include few species. They provide precision in measurement but the general significance of each mechanism at a global scale is more difficult to study because inevitably datasets are small. In contrast, as outlined above, the ‘functional trait’ approach can quickly generate large datasets for whole floras with each species defined in many contrasted ecological dimensions. As in other comparative approaches the ‘functional trait’ approach has a strong potential for generality. ‘The collection and comparison of standardized information …… follows closely the philosophies prevailing in the physical sciences. Perhaps the most obvious of these is the role played by the Periodic Table of the Elements in classifying, analysing, and even predicting, the structure and properties of chemical elements and compounds’ [49]. Why has the ‘functional trait’ approach had so little impact on understanding the role of climate on plant distribution? The failure appears to stem from the consistent use of a restricted and ‘climatically inappropriate’ set of traits. Traits linked to ‘mechanistic’ climate-related studies are conspicuously absent from ‘functional’ analyses both in studies describing general global patterns of specialization [12,13] and in those with a more climatic focus [36,37]. A plethora of potentially important climate-related traits still require further investigation. With interdisciplinary collaboration, the ‘functional trait’ approach has the potential to add important elements of generality and utility to the current pioneering ‘mechanistic’ studies …. provided, of course, that suitable, easily- and rapidly-measurable traits can be borrowed from the existing raft of ‘mechanistic’ studies.

### Objectives

**Fig 1:**
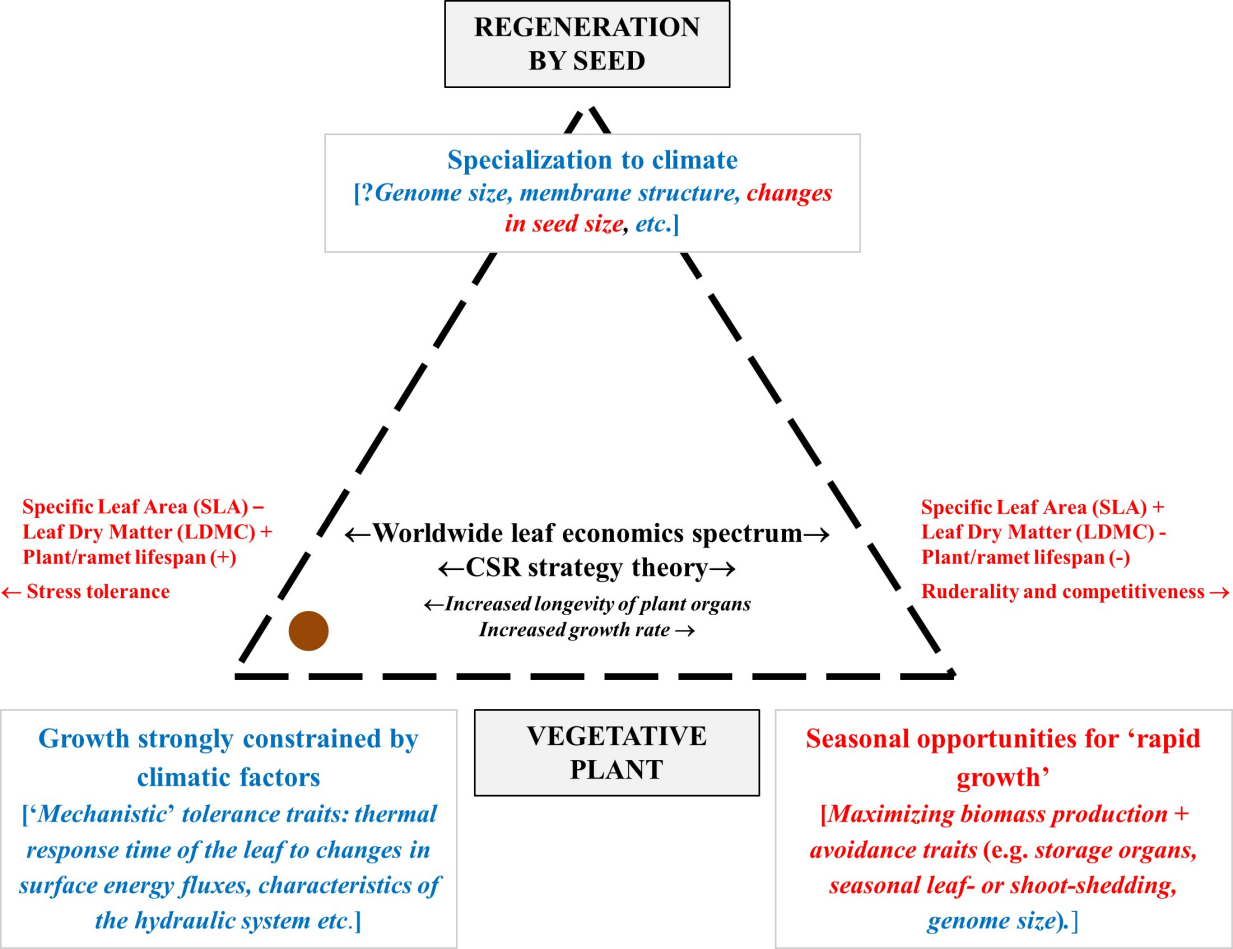
Predicted broad trends between plant traits and response to climate. ‘Mechanistic’, relating particularly to specializations for surviving harsh climates, and ‘functional’ traits sensu Broderibb [47], that may optimize growth during other periods, are coloured blue and red respectively. ● identifies the putative position of Olea designated with a orange color.

Our trait-based study will focus upon climate and, in particular, oleaster, or wild olive, growing along a climatic gradient in Mediterranean Morocco. Both species and region are of climatic relevance and interest. Climate change is predicted to increase the geographical range of the olive [50]. Equally, the Mediterranean region is both a biodiversity hotspot [51] and under severe threat from climate change [52,53]. Specifically, variation in trait expression will be explored between and within nineteen populations of oleaster from climatically-contrasted habitats. The traits to be measured fall into two groups. The first group comprises ‘functional traits’, attributes that identify the ‘worldwide leaf economics spectrum’ and plant size. These traits are essentially those routinely measured in ‘functional’ studies. The second group includes ‘mechanistic’ traits linked to climate by physiological studies but not currently used in most ‘functional trait’ studies. Of necessity, we will concentrate attention on ‘mechanistic’ traits that may be measured rapidly. We shall assess both individually and in combination, which traits pattern more exactly with climatic variables, (a) ‘functional’ or (b) ‘ecophysiological’ traits. In this context, we suggest that field responses to climate has two components (Fig 1). First, ‘functional’ strategies relating to the ‘leaf economics spectrum’ may be expected to optimize resource acquisition and growth during less climatically-severe periods. Secondly, ‘mechanistic’ strategies involving ‘ecophysiological traits’ linked to climate by physiological studies will confer tolerance of climatic extremes. Since oleaster is both slow-growing and long-lived tree, survival of climatic extremes will be key. Accordingly, our specific hypothesis is that, for oleaster, ‘ecophysiological traits’ little-used in trait-based plant ecology will pattern with climatic variables more effectively than commonly-used ‘functional traits’. More generally, we suspect that ‘functional’ trait-based plant ecology will have little impact on climate change research until it routinely includes a ‘mechanistic’ dimension to its trait measurements and analyses.

## Materials and methods

### Study species

Oleaster (*Olea europaea* subsp. *europaea* var. *sylvestris* (Miller) Lehr) is the wild progenitor of the Mediterranean cultivated olive (*Olea europaea* L. subsp. *europaea* var. *europaea*) (Besnard et al. [54] for review). It is an evergreen, sclerophyllous, long-lived wind-pollinated tree [55–59]. Oleaster is a characteristic component of the natural Mediterranean vegetation [60,61] and its presence may be traced back to at least the last glaciation period [61–63]. It is considered a bio-indicator of the Mediterranean climate [61,64–67] and arguably the most emblematic and iconic tree of the Mediterranean Basin [54]. In Morocco climax vegetation with oleaster extends over several Mediterranean bioclimates from humid to arid along a north-south latitudinal gradient and oleaster appears eliminated mainly by the cold (maximum altitude between 1000 and 1600 m) and extreme aridity [55]. Because of its importance, (a) in climate studies, as a bio-indicator, and (b) in studies of ecosystem function, as a major component of the vegetation, oleaster is an ideal species for studying traits along a climatic gradient.

### Study areas

**Fig 2:**
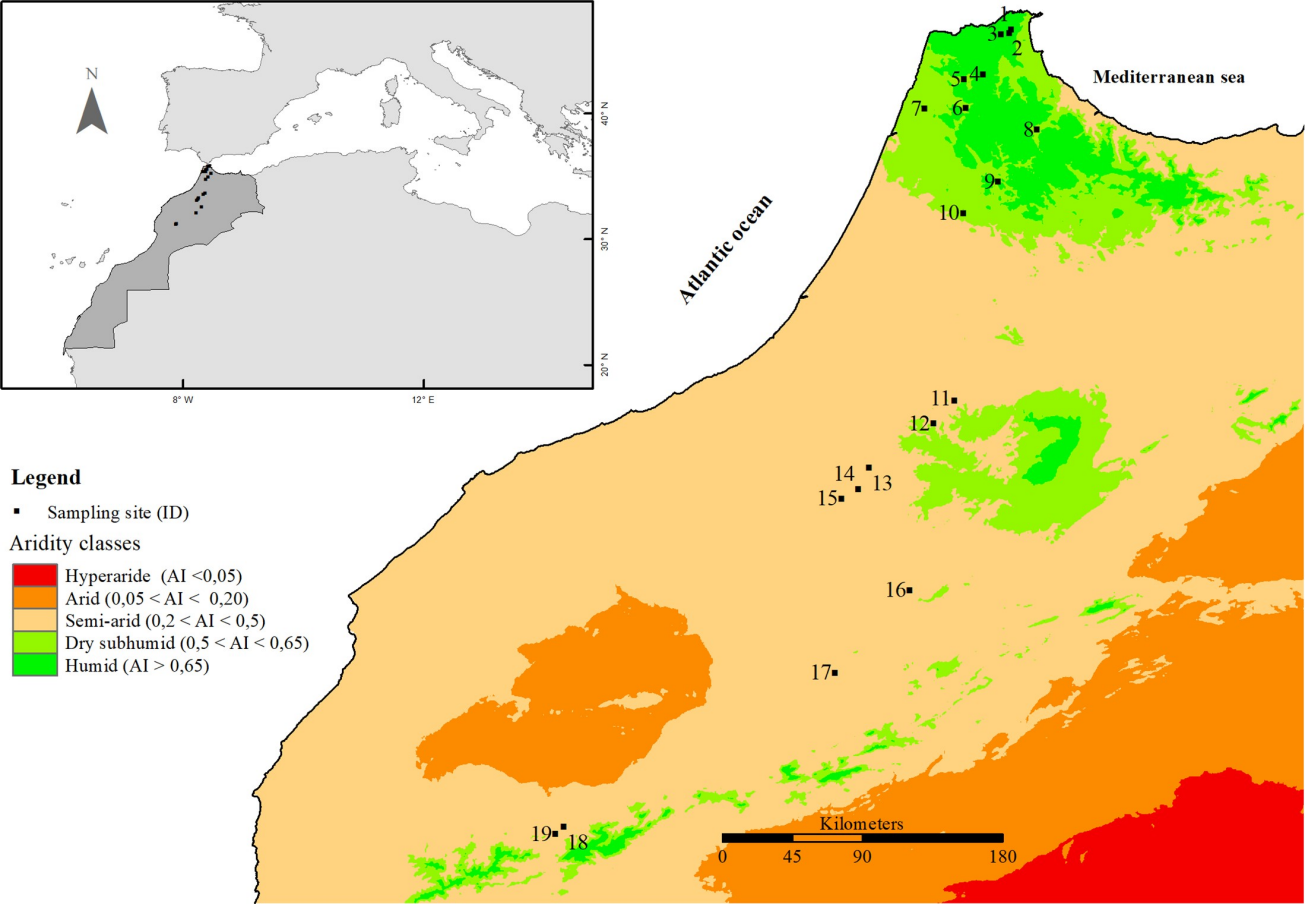
The geographical locations of sampling sites across Morocco with contours for Aridity index generated with Open Quantum GIS V. 2.12.3-Lyon software [79]. Site numbers accord with Table 1.

The field context was provided by a 600 km latitudinal transect of Morocco that includes a wide range of habitats and vegetation types across three bioclimatic zones (humid, subhumid and semi-arid) within Morocco (Fig 2) [68–73]. We chose nineteen representative woodland and scrub sites each with contrasted climatic, geographical and phytoecological characteristics (Table 1). The climate of each site was defined using five variables: mean annual temperature (MAT,°C), minimum temperature of the coldest month (MTCM, °C), and the mean annual precipitation (MAP, mm), all extracted from the Worldclim database, at a resolution of 30 arc’s (~1 km^2^) [74] plus the mean annual evapotranspiration (PET, mm/year) and the global aridity index (AI, unitless, calculated as MAP/PET) from, respectively, the PET database and the CGIAR Global Aridity database [75,76]. The range of climate associated with our sites (Table 1) is greater than changes to climatic variables predicted by Gibelin and Déqué [77] and Polade et al. [78] making our study area more relevant to climate change research.

**Table 1:** Climatic, geographic and phytoecological characteristics of sites sampled.

Site names	Latitude (°)	Longitude (°)	Altitude (m)	MAT (°C)	MTCM (°C)	MAP (mm/year)	AI	Vegetation series (types)	Phytoecological associations
**(A) Humid climate**									
1. Tlat Taghramt Med	-5.458	35.807	364	16.5	5.8	808	0.72	Kermes oak Thermo-mediterranean serie	*Rusco hypophyllii-Quercetum cocciferae* [69]
2. Tlat Taghramt	-5.468	35.789	293	16.9	6.3	801	0.71		
3. Ksar Sghir	-5.515	35.783	255	17.5	7.1	802	0.71		
**(B) Dry subhumid climate**									
4. Bni Harchin	-5.620	35.551	150	18.0	7.3	779	0.65	Oleaster and carob Thermo-mediterranean serie	*Tamo communis-Oleetum sylvestris* [69]
5. Dar Chaoui	-5.730	35.521	64	18.1	7.7	751	0.63		
6. Bni Arous	-5.718	35.357	90	18.1	6.5	781	0.62		
7. Tnin Sidi Yamani	-5.958	35.353	126	17.7	6.1	765	0.61		
8. Dar Akoubaa	-5.310	35.231	322	17.7	5.6	774	0.61		
9. Ouezzene	-5.533	34.930	137	18.7	5.9	814	0.60		
10. Mesmouda	-5.734	34.750	196	18.0	5.0	805	0.59		
**(C) Semi-arid climate**									
11. Ras Ejery	-5.785	33.667	589	16.3	2.8	621	0.44	Barbary thuya mesophil serie	*Coronilla viminalis-Tetraclinetum articulatae* [73]
12. Bouqachmir	-5.906	33.536	582	16.8	3.2	582	0.40		
13. Moulay Bouazza	-6.436	33.102	680	17.3	2.8	482	0.31	Holm oak Thermo-mediterranean serie	*Smilaci mauritanicae-Quercetum rotundifoliae* [73]
14. Sebt Ait-Rahou	-6.280	33.280	745	16.6	2.2	545	0.31	Barbary thuya mesophil serie	*Coronilla viminalis-Tetraclinetum articulatae* [73]
15. Oulad Aissa	-6.341	33.158	523	18.1	4.0	472	0.37		
16. El Ksiba	-6.044	32.575	900	16.6	1.4	721	0.46	Phoenicean juniper and barbary thuya serie	*Querco rotundifoliae-Tetraclinetum articulatae* [80]
17. Bin Ouidane	-6.475	32.098	946	17.1	1.8	514	0.32		*Polygalo balansae- Tetraclinetum articulatae* [73]
18. Asni	-8.041	31.208	953	15.6	0.2	420	0.28		*Lavandulo dentatae-Tetraclinetum articulatae* [80]
19. Ouirgane	-8.092	31.167	919	16.5	1.1	377	0.26		

Climatic variables, abstracted from Worldclim database [74] and the CGIAR Global Aridity and PET database [76], are abbreviated as follows: MAT (°C), mean annual temperature; MTCM (°C), mean temperature of the coldest month; MAP (mm), mean annual precipitation; AI: aridity index, calculated as MAP/PET (PET, potential evapotranspiration) with low values indicate more arid habitat. Vegetation type relates to Benabid and Fennane [71] and phytoecological association to Barbéro et al. [73], Benabid [69] and Fennane [80].

### Field sampling

Sampling in the field requires no authorization; the study sites are located in natural public areas outside protected natural areas. The species studied (*Olea europaea* subsp. *europea sylvestris* (Miller) Lehr) is not a rare or protected species.

At each site, ten healthy oleaster trees were randomly selected from within of a 200m^2^ area during the spring (April to May) of 2016. The diameter at breast height (DBH) and the vegetative height (H) of each tree were recorded as proscribed by Bonham [81]. Subsequently, relative chlorophyll content (CHL, SPAD units) was measured using a SPAD 502 plus chlorophyll meter and following the protocols of Cornelissen et al. [82], thirty healthy, mature leaves were collected at random from the south (sun-exposed) side of the upper canopy and returned to the laboratory on the day of sampling in a cool, hydrated condition.

### Leaf traits

Seventeen plant traits were measured (Table 2). Six were ‘functional’ traits, relating to size or to the ‘leaf economics spectrum’, of the type more traditionally used in trait-based ecological studies. We are trialing a further six ‘mechanistic’ traits identified from the physiological literature as of possible relevance in climate studies. Additionally, five ‘intermediate’ traits were included. These were potentially relevant to both resource capture and to climate. Traits were further categorized into four inexact groupings relating to what was being measured: (1) ‘ecophysiological’, both function and measurement relating to one component of leaf form and function (e.g. stomatal density (DS)); (2) ‘morphological size and shape’, both function and measurement relating to the whole leaf (e.g. leaf area (LA)), (3) ‘structural allocation’, function estimated from interacting properties of the whole leaf (e.g. specific leaf area (SLA)) and (4) whole plant (e.g. plant height, m; H). Group 1 is analogous to ‘mechanistic’ in so far as there is a relatively precise and direct connection between how the trait functions and what was measured. Also groups 3–4 equate to ‘functional’ traits and group 2 may be viewed as intermediate, with impacts upon both growth and water use efficiency. As a matter of policy, we have opted to concentrate on traits that may be measured quickly and easily. Such traits may be more readily incorporated into trait-based ecological studies.

**Table 2:** Leaf traits studied inexactly grouped according to putative function and reasons for their use.

Trait grouping	Traits	Abbr.	Unit	Putative functional role	References
Ecophysiological (within leaf)	Relative chlorophyll content^m^	CHL	SPAD unit	Photosynthetic rate and leaf life span	[88]
	Stomatal density^m^	DS	no. of stomata abaxial surface mm^-2^	Stomatal conductance and water balance	[89] [90] [91] [92]
	Leaf water content^m^	LWC	g	Water balance	
	Specific leaf water content^m^	SLWC	g H_2_O cm^-2^	Water balance	[85]
	Leaf thickness^f^^,^^m^	LT	mm	Resource acquisition and water balance	[93] [94] [95] [96]
Morphological size and shape (whole leaf)	Leaf area^f^^,^^m^	LA	cm^2^	Resource capture, growth rate and water balance	[93][97] [98] [99] [12] [13]
	Length^f^^,^^m^	LL	cm	Light capture, resource capture and growth rate	[93] [100] [101] [99] [87]
	Width^f^^,^^m^	LW	cm		
	Length at maximum width^f^^,^^m^	LL_max_	cm		
	Length:width ratio^m^	LL/LW		Light capture and thermoregulation	[86] [87]
	Length:LPL ratio^m^	LL/LL_max_		Light capture and thermoregulation	
Structural allocation (whole leaf)	Leaf fresh mass^f^	LWM	g	Resource acquisition	[102] [103]
	Leaf dry mass^f^	LDM	g	Resource acquisition	
	Specific leaf area^f^	SLA	cm^2^ g^-1^	Resource capture, water balance and growth rate	[102] [104] [105] [9] [106]
	Leaf dry matter content^f^	LDMC	mg g^-1^	Physical resistance, stress tolerance and growth rate	[107] [5] [103] [82]
Plant size (whole plant)	Plant height^f^	H	m	Light capture, competition rate, stress tolerance and growth rate	[107] [5] [108]
	Diameter at breast height^f^	DBH	cm	Resource capture, stress tolerance and growth rate	[109]

^m^ denotes ‘mechanistic’ traits, whose relevance has been identified directly from physiological studies, and

^f^ ‘functional’ traits relating to size or to the ‘leaf economics spectrum’. Traits that span both groupings are designated as ^f,m^.

For each leaf trait there were 30 replicate leaves tree^-1^ x 10 trees site^-1^ x 19 sites, making a total of 5700 replicate leaves. Trichomes were removed from the abaxial leaf surface of *Olea*, a hypostomatous species and impressions made using clear nail polish [83,84]. Subsequently, stomatal density (DS, no. of stomata mm^-2^) was measured from four separate areas of the abaxial surface of the leaf at 400x magnification (Olympus BX43). Additional laboratory- measured traits were–*group 1*: leaf water content (LWC, g; LWM–LDM), specific leaf water content (SLWC, g cm^-2^; (LWM–LDM)/LA) [85]), leaf thickness (LT, mm, using a precision micrometer (Mitutoyo, 0,01-25mm)); *group 2*: leaf area (LA, cm^2^), length (LL, cm), width (LW, cm), length of the broadest leaf (LL_max_, cm), two estimates of leaf shape length:width ratio (LL/LW) and length: longest length ratio (LL/LL_max_), [86,87]; *group 3*: leaf fresh mass (LWM, mg), leaf dry mass (LDM, mg), specific leaf area (SLA, cm^2^ g^-1^; LA/LDM); leaf dry matter content (LDMC mg g^-1^, LDM/LWM). In addition, the functional syndrome, CSR-strategy, was calculated from leaf traits by the method of Pierce et al. [4].

### Data analysis

After checking frequency distributions for normality and variance heterogeneity, it was necessary to log-transform trait values prior to analysis. Subsequently, one-way ANOVA with Tukey HSD *post hoc* tests, Pearson correlations and principal component analyses (PCA) were undertaken for inter-population comparisons of trait values and of coefficient of variation (CV) estimates using the open source statistical environment R 3.3.3 [110].

**Fig 3:**
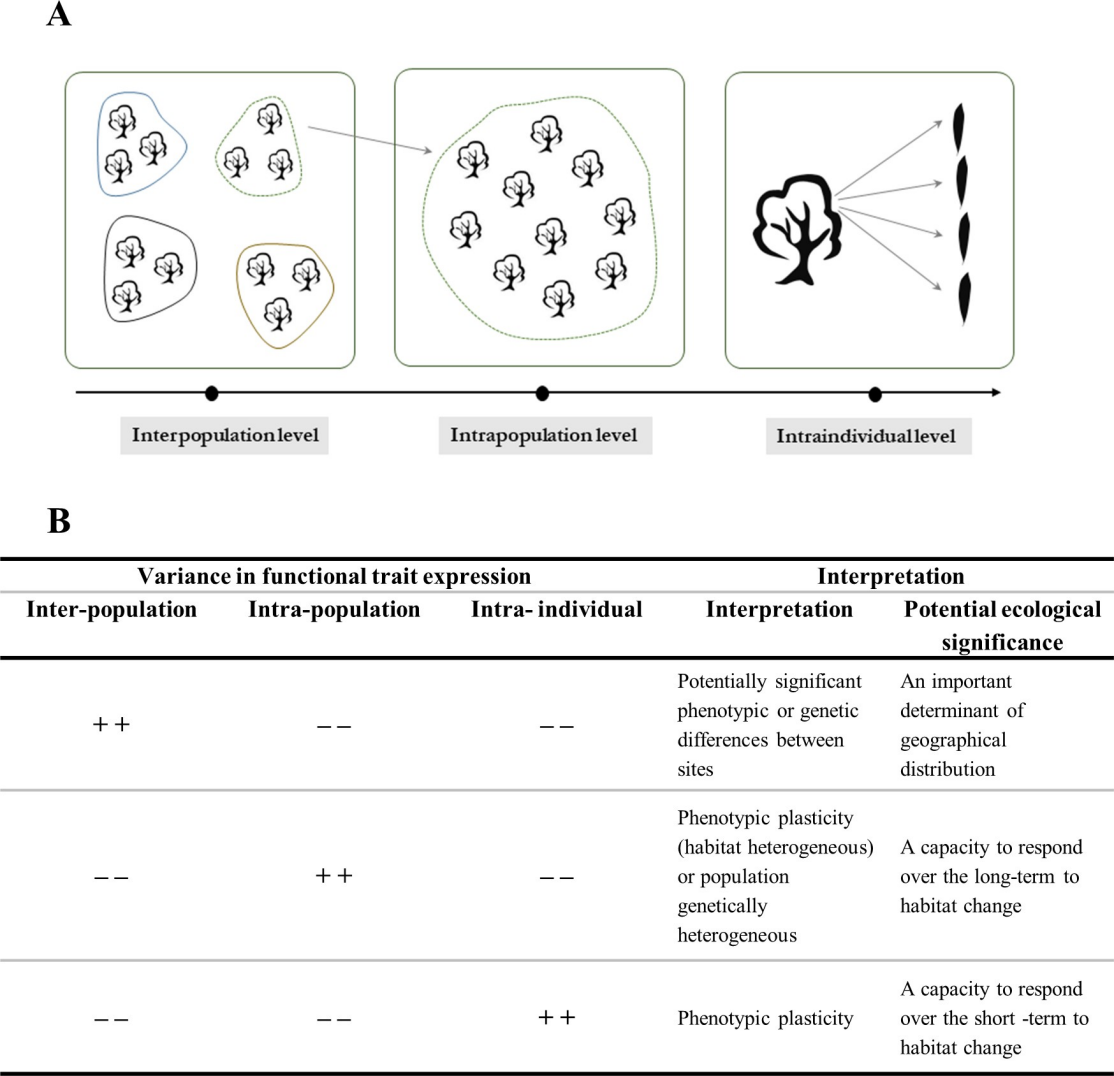
(A) The hierarchical sampling design (19 sites x 10 trees site^-1^ x 30 leaves tree^-1^) and (B) the possible ecological significance of contrasted patterns of distribution.

Intraspecific variation of the 5700 replicates for each leaf trait was explored across three levels of spatial and organizational scales: 1) ‘regional’, between different sites; 2) ‘population’, between different trees at same site, and 3) ‘individual’, between leaves on the same tree (Fig 3). Here, the coefficient of variation (CV), calculated by the formula CV (%) = standard deviation_trait_/mean_trait_ x 100 [111], evaluates the amplitude of trait variability [112,113]. This is particularly true where there is a proportionality between the mean and standard deviation of the distribution [114]. The ‘varcomp’ function in the ‘ape’ package extracted these variance components [115] and to quantify the extent and importance of variance across regional, population and individual scales, a general linear mixed model was fitted using the restricted maximum likelihood (RMEL) method, a nested ANOVA with random effects [22]. Subsequently, the Canberra metric (CM) calculated as: CM = (CV_max_−CV_min_)/(CV_max_ + CV_min_) tested for significant differences between the two coefficients of variation [116–118]. When the value of CM is higher than 0.1, differences are considered significant.

## Results

### Ecological relevance of coefficients of variation (CV) values

Mean and standard deviation for all measured leaf traits differed markedly in their amplitude of variation. Nevertheless, apart from DS, both were significantly correlated (X *v σ* calculations in Tables 3, 4, 5 and 6, S1 Table). Moreover, CV values generally did not vary in the same direction as the variance ratio, *F*. Thus, for example, LT had a low CV value (21%) but high *F* value and appeared strongly discriminant between populations (Table 3). Also, values for the Canberra metric (0.19 to 0.69; S1 Table) exceeded the 0.1 threshold for statistical significance. Our more extreme inter-population differences in CV for each traits may be viewed as both statistically and potentially ecologically significant.

**Table 3:** Mean traits ± 95% confidence limits of ‘ecophysiological’ leaf traits.

Population	CHL^m^	CV%	DS^m^ (n/mm_2_)	CV%	LWC^m^	CV%	SLWC^m^ (g H_2_O cm^-2^)	CV%	LT^f,m^ (mm)	CV%
(A) Humid climate										
1. Tla Taghramt Med	72.9 ± 1.1^c,d^	13.3	505 ± 4^f^	7.3	0.089 ± 0.003^c,d^	34.3	0.031 ± 0.001^c,d^	40.6	0.36 ± 0.01 ^h,i^	11.1
2. Tla Taghramt	58.9 ± 1.3^k^	20.0	523 ± 5^e^	9.2	0.090 ± 0.004^c,d^	36.8	0.025 ± 0.001^e,f,g^	27.3	0.31 ± 0.01^k^	15.5
3. Ksar Sghir	70.7 ± 0.5^d,e,f^	6.1	454 ± 4^h^	7.7	0.062 ± 0.003^g,h^	37.3	0.028 ± 0.001^c,d,e^	34.8	0.44 ± 0.01^c^	12.4
(B) Dry subhumid climate										
4. Bni Harchin	69.9 ± 1.2^e,f,g^	15.3	276 ± 8^m^	26.3	0.113 ± 0.005^b^	39.7	**0.037 ± 0.002**^**b**^	42.0	0.37 ± 0.01 ^h,i^	17.5
5. Dar Chaoui	**77.8 ± 2.6**^**a**^	30.1	417 ± 5^j^	11.1	0.059 ± 0.003^g,h^	45.7	0.024 ± 0.001 ^e,f,g^	47.5	0.39 ± 0.01^e,f,g^	10.5
6.Bni Arous	66.8 ± 1.0^h,i^	13.4	364 ± 6^l^	15.7	0.098 ± 0.006^c^	53.6	**0.040 ± 0.002**^**b**^	44.1	0.38 ± 0.01^f,g,h^	11.1
7. Tnin Sidi Yamani	61.8 ± 1.0^j^	14.6	390 ± 8^k^	17.2	**0.115 ± 0.006**^**b**^	46.8	0.031 ± 0.002^c,d^	54.6	0.37 ± 0.01 ^h,i^	11.5
8. Dar Akoubaa	**76.5 ± 1.1**^**a,b**^	12.6	359 ± 6^l^	14.3	**0.120 ± 0.005**^**b**^	37.4	0.031 ± 0.001^c^	34.2	**0.48 ± 0.01**^**b**^	9.2
9. Ouezzene	67.7 ± 0.8^g,h,i^	10.6	439 ± 6^h,i^	12.0	0.058 ± 0.003^g,h,i^	50.6	0.026 ± 0.001^d,e,f,g^	42.0	0.40 ± 0.01^d,e,f^	10.1
10. Mesmouda	65.6 ± 1.1^i^	14.8	404 ± 5^j,k^	11.0	0.085 ± 0.005^d^	47.2	0.030 ± 0.001^c,d,e^	41.3	0.44 ± 0.00^c^	8.3
(C) Semi-arid climate										
11. Ras Ejery	72.2 ± 0.8^c,d,e^	9.4	561 ± 7^d^	10.8	0.063 ± 0.003^f,g^	40.8	0.021 ± 0.001^g^	25.3	0.37 ± 0.01^h,i^	14.4
12. Bouqachmir	**76.7 ± 0.7**^**a**^	8.2	362 ± 5^l^	12.2	0.081 ± 0.005 ^d,e^	51.8	0.025 ± 0.003 ^e,f,g^	92.1	0.41 ± 0.01^d^	10.2
13. Moulay Bouazza	**77.4 ± 0.9**^**a**^	10.8	**603 ± 6**^**b**^	13.5	**0.388 ± 0.005**^**a**^	12.4	**0.167 ± 0.006**^**a**^	33.8	**0.60 ± 0.019**^**a**^	27.8
14. Sebt Ait-Rahou	73.8 ± 0.8^b,c^	9.0	577 ± 7^c^	10.3	0.083 ± 0.003^d,e^	37.0	0.023 ± 0.001^f,g^	27.6	0.38 ± 0.001^g,h,i^	12.4
15. Oulad Aissa	71.4 ± 0.9^c,d,e,f^	10.7	**603 ± 6**^**b**^	9.3	0.055 ± 0.002^g,h,i^	35.0	0.026 ± 0.001 ^d,e,f,g^	26.7	0.34 ± 0.01^j^	12.3
16. El Ksiba	69.7 ± 0.9^e,f,g^	11.1	481 ± 9^g^	15.9	0.052 ± 0.002^h,i^	35.9	0.022 ± 0.001^f,g^	35.5	0.40 ± 0.01^d,e^	11.2
17. Bin Ouidane	62.2 ± 0.7^j^	10.5	521 ± 6^e^	10.5	0.049 ± 0.002^i^	35.0	0.026 ± 0.001^c,d,e,f,g^	27.7	0.43 ± 0.01^c^	9.5
18. Asni	68.6 ± 0.8^f,g,h^	10.1	**621 ± 6**^**a**^	8.9	0.073 ± 0.005^e,f^	66.5	0.027 ± 0.003^c,d,e,f^	85.1	0.44 ± 0.00^c^	8.8
19. Ouirgane	69.8 ± 1.0^e,f,g^	12.9	438 ± 6^i^	12.8	0.080 ± 0.004 ^d,e^	38.7	0.021 ± 0.001^g^	29.8	0.36 ± 0.01^i^	17.9
Mean	70.0 ± 0.3	15.8	462.3 ± 3	23.6	0.092 ± 0.002	85.4	0.034 ± 0.001	105.51	0.40 ± 0.00	20.9
X *v σ* (*r*)	0.300***		0.088 ns		0.428***		0.772***		0.598***	
ANOVA *F*_*19*,*5699*_	91.7 ***		947.1 ***		1242.2 ***		962.4 ***		328.3 ***	

**Table 4:** Mean traits ± 95% confidence limits of ‘morphological size and shape’ leaf traits.

Population	LA^f,m^ (cm^2^)	CV%	LL^f,m^ (cm)	CV%	LW ^f,m^ (cm)	CV%	LL_max_^f,m^ (cm)	CV%	LL/LW^m^	CV%	LL/LL_max_^m^	CV%
(A) Humid climate												
1. Tla Taghramt Med	3.04 ± 0.10^d,e^	29.5	3.49 ± 0.08^h.i^	19.9	1.25 ± 0.02^b^	17.4	2.20 ± 0.07^e,f^	27.2	2.84 ± 0.07^j^	20.7	1.65 ± 0.05 ^e,f,g,h^	24.3
2. Tla Taghramt	**3.71 ± 0.15**^**b**^	35.6	3.81 ± 0.09^e,f,g^	22.0	**1.37 ± 0.03**^**a**^	19.7	2.60 ± 0.08^c,d^	26.5	2.80 ± 0.05^j^	17.0	1.51 ± 0.03^i^	19.8
3. Ksar Sghir	2.27 ± 0.08^h,i^	31.8	3.10 ± 0.07^k^	21.1	1.09 ± 0.02^d,e^	14.0	1.97 ± 0.08 ^h,i^	33.9	2.88 ± 0.07^i.j^	22.6	1.73 ± 0.08^d,e^	39.1
(B) Dry subhumid climate												
4. Bni Harchin	3.33 ± 0.14^c,d^	37.8	3.64 ± 0.11^g,h^	26.4	**1.37 ± 0.03**^**a**^	19.8	2.26 ± 0.08^e^	31.2	2.68 ± 0.07^j^	22.8	1.68 ± 0.05^e,f,g^	26.0
5. Dar Chaoui	2.57 ± 0.09^g,h^	31.0	3.25 ± 0.08 ^i,j,k^	21.5	1.15 ± 0.02^c,d^	18.1	1.98 ± 0.05 ^h,i^	22.2	2.90 ± 0.08^i,j^	23.5	1.66 ± 0.03 ^e,f.g,h^	14.1
6.Bni Arous	2.45 ± 0.11^g,h,i^	40.9	3.33 ± 0.11^i,j,k^	29.8	1.08 ± 0.02^e^	18.4	2.18 ± 0.10^e,f,g^	40.8	3.10 ± 0.08^h,i^	24.3	1.64 ± 0.05 ^e,f,g,h^	26.2
7. Tnin Sidi Yamani	**3.99 ± 0.15**^**a,b**^	32.5	4.34 ± 0.08^c^	16.1	**1.35 ± 0.03**^**a**^	21.0	**2.69 ± 0.07**^**b,c**^	22.7	3.30 ± 0.07^g,h^	18.2	1.67 ± 0.04^e,f,g,h^	23.2
8. Dar Akoubaa	**4.08 ± 0.19**^**a**^	42.1	**5.38 ± 0.15**^**a**^	25.1	1.24 ± 0.04^b^	27.3	**3.55 ± 0.13**^**a**^	33.0	**4.47 ± 0.11**^**a**^	22.0	1.59 ± 0.05^g,h,i^	25.3
9. Ouezzene	2,29 ± 0.09^h,i^	33.4	3.38 ± 0.08^i,j^	20.0	0.98 ± 0.02^f,g^	17.9	1.99 ± 0.08^h,i^	34.6	3.49 ± 0.07^d,e,f,g^	19.0	1.86 ± 0.07^b,c^	34.5
10. Mesmouda	2.89 ± 0.09^e,f^	28.6	3.98 ± 0.08^d,e^	17.5	1.13 ± 0.03^d,e^	20.8	2.56 ± 0.07^c,d^	24.2	3.62 ± 0.09^c,d,e^	22.5	1.61 ± 0.04^f,g,h^	22.5
(C) Semi-arid climate												
11. Ras Ejery	2.98 ± 0.11^e,f^	32.2	3.89 ± 0.08^e,f^	17.7	1.15 ± 0.03^c,d^	19.3	1.90 ± 0.04 ^i^	18.9	3.43 ± 0.07^e,f,g^	17.3	**2.06 ± 0.03**^**a**^	11.8
12. Bouqachmir	3.38 ± 0.13^c^	34.8	4.19 ± 0.08^c,d^	16.8	1.20 ± 0.03^b,c^	21.0	2.23 ± 0.05^e,f^	19.7	3.57 ± 0.06^d,e,f^	14.7	**1.90 ± 0.02**^**b**^	10.9
13. Moulay Bouazza	2.55 ± 0.09^g,h^	31.5	3.78 ± 0.08^e,f,g^	19.7	1.01 ± 0.02^f^	18.2	2.11 ± 0.04^e,f,g,h^	17.8	**3.81 ± 0.09**^**b,c**^	20.1	1.80 ± 0.02^c,d^	8.7
14. Sebt Ait-Rahou	3.70 ± 0.14^b^	33.1	4.26 ± 0.08^c^	16.7	1.31 ± 0.03^a^	21.4	2.49 ± 0.05^d^	19.2	3.33 ± 0.07^g^	19.6	1.73 ± 0.02^d,e^	12.0
15. Oulad Aissa	2.19 ± 0.07^i,j^	29.9	3.26 ± 0.09 ^i,j,k^	24.1	0.99 ± 0.02^f,g^	16.5	2.01 ± 0.06^g,h,i^	24.3	3.37 ± 0.11^f,g^	27.8	1.63 ± 0.02 ^e,f,g,h^	9.2
16. El Ksiba	2.46 ± 0.08^g,h,i^	28.5	3.66 ± 0.08^f,g,h^	19.3	1.01 ± 0.02^f^	16.7	1.96 ± 0.04 ^h,i^	19.0	**3,69 ± 0.08**^**b,c,d**^	20.2	**1.88 ± 0.02**^**b,c**^	10.6
17. Bin Ouidane	1.92 ± 0.07^j^	33.1	3.19 ± 0.06^j,k^	17.8	0.94 ± 0.02^g^	18.9	2.06± 0.03^f,g,h,i^	12.2	3.56 ± 0.12^d,e,f^	29.3	1.57 ± 0.03^h,i^	18.7
18. Asni	2.70 ± 0.12^f,g^	39.1	4.19 ± 0.15^c,d^	31.2	0.96 ± 0.02^f,g^	18.6	2.10 ± 0.08^e,f,g,h^	31.6	**4.33 ± 0.12**^**a**^	24.5	**2.01 ± 0.02**^**a**^	9.5
19. Ouirgane	**3.96 ± 0.16**^**a,b**^	35.1	**4.73 ± 0.12**^**b**^	22.9	1.24 ± 0.03^b^	18.8	**2.81 ± 0.08**^**b**^	25.3	**3.86 ± 0.10**^**b**^	21.7	1.70 ± 0.02^d,e,f^	11.9
Mean	2.92 ± 0.03	41.7	3.39 ± 0.03	26.1	1.14 ± 0.01	14.0	2.27 ± 0.02	32.3	3.39 ± 0.02	26.1	1.73 ± 0.01	22.1
X *v σ* (*r*)	0.792***		0.602***		0.564***		0.525***		0.610***		0.316***	
ANOVA *F*_*19*,*5699*_	124.1 ***		143.4 ***		122.3 ***		127.8 ***		128.8 ***		52.8 ***	

**Table 5:** Mean traits ± 95% confidence limits of ‘structural allocation’ leaf traits.

Population	LWM^f^ (g)	CV%	LDM^f^ (g)	CV%	SLA^f^ (cm^2^ g^-1^)	CV%	LDMC^f^ (g. mg^-1^)	CV%
(A) Humid climate								
1. Tla Taghramt Med	0.135 ± 0.004^c,d,e^	28.5	0.046 ± 0.002^f,g,h^	33.1	70.0 ± 2.7^d^	34.0	347.2 ± 9.8^g,h^	25.0
2. Tla Taghramt	0.130 ± 0.005^d,e^	34.0	0.041 ± 0.002^h,i,j^	44.4	**96.9 ± 3.7**^**a**^	33.4	316.8 ± 9.6^h,i^	26.7
3. Ksar Sghir	0.107 ± 0.004^f^	32.5	0.046 ± 0.002^f,g,h,i^	38.6	52.6 ± 2.0^h,i,j^	32.8	429.8 ± 11.4 ^b,c^	23.3
(B) Dry subhumid climate								
4. Bni Harchin	**0.164 ± 0.007**^**b**^	36.6	0.051 ± 0.003^d,e,f^	53.3	**78.6 ± 4.6**^**b,c**^	52.5	311.0 ± 13.56^i^	38.6
5. Dar Chaoui	0.102 ± 0.004^f^	32.2	0.043 ± 0.002^g,h,i^	34.6	62.7 ± 2.0^e,f^	27.7	433.9 ± 14.3 ^b,c^	29.0
6.Bni Arous	0.138 ± 0.007^c,d^	43.2	0.041 ± 0.002 ^h,i,j^	38.3	63.0 ± 2.5^e,f^	34.1	323.9 ± 13.6^h,i^	35.9
7. Tnin Sidi Yamani	**0.174 ± 0.007**^**b**^	34.8	**0.059 ± 0.003**^**b,c**^	37.3	72.1 ± 3.6^c,d^	42.4	364.0 ± 16.7^f,g^	38.7
8. Dar Akoubaa	**0.214 ± 0.008**^**a**^	34.3	**0.096 ± 0.005**^**a**^	46.6	47.2 ± 3.0^j^	54.1	441.1 ± 14.1^b,c^	27.0
9. Ouezzene	0.097 ± 0.004^f,g^	40.8	0.041 ± 0.002^g,h,i,j^	38.2	59.3 ± 1.7^f,g,h^	63.6	429.5 ± 14.5 ^b,c^	28.4
10. Mesmouda	0.147 ± 0.005^c^	32.8	**0.064 ± 0.002**^**b**^	29.0	47.6 ± 1.9^i,j^	35.2	**456.3 ± 15.8**^**a,b**^	29.9
(C) Semi-arid climate								
11. Ras Ejery	0.104 ± 0.004^f^	33.4	0.041 ± 0.001^g,h,i,j^	29.6	74.1 ± 2.3^c,d^	27.2	409.6 ± 10.1^c,d,e^	21.8
12. Bouqachmir	0.137 ± 0.005^c,d^	32.7	0.057 ± 0.002^c,d^	30.9	60.0 ± 1.7^f,g^	24.3	425.9 ± 9.1 ^b,c,e^	18.9
13. Moulay Bouazza	0.103 ± 0.004^f^	31.9	0.040 ± 0.002^i,j^	40.6	67.7 ± 2.4^d,e^	37.3	394.0 ± 11.2^e,f^	25.5
14. Sebt Ait-Rahou	0.139 ± 0.006^c,d^	36.6	0.055 ± 0.003^c,d,e^	45.0	72.7 ± 2.4^c,d^	29.3	396.6 ± 10.6^d,e^	23.5
15. Oulad Aissa	0.083 ± 0.003^h^	30.5	0.029 ± 0.001^k^	38.3	**83.1 ± 3.2**^**b**^	33.7	344.5 ± 10.5^g,h^	26.5
16. El Ksiba	0.099 ± 0.003^f^	27.7	0.047 ± 0.002^f,g^	31.3	54.5 ± 1.9^g,h,i^	31.0	**476.9 ± 11.3**^**a**^	21.0
17. Bin Ouidane	0.085 ± 0.003^g,h^	31.4	0.036 ± 0.001^j^	35.5	54.8 ± 1.5^g,h^	24.6	430.4 ± 10.0^b,c^	20.4
18. Asni	0.123 ± 0.006^e^	45.2	0.050 ± 0.002^e,f^	35.6	56.8 ± 2.80^f,g,h^	43.2	438.3 ± 15.8 ^b,c^	31.7
19. Ouirgane	0.144 ± 0.006^c^	35.3	**0.064 ± 0.003**^**b**^	44.1	70.3 ± 3.7^d^	46.9	438.3 ± 13.0 ^b,c^	26.2
Mean	0.125 ± 0.001	44.1	0.049 ± 0.001	49.9	65.3 ± 0.7	40.9	406.3 ± 3.1	29.8
X *v σ* (*r*)	0.671***		0.795***		0.635***		0.364***	
ANOVA *F*_*19*,*5699*_	154.4 ***		141.3 ***		82.9 ***		63.3 ***	

**Table 6:** Mean traits ± 95% confidence limits whole plant traits (i) and syndromes (ii).

Population	(i) H^f^	CV%	DBH^f^	CV%	(ii) C% (*CSR strategy*)
(A) Humid climate					
1. Tla Taghramt Med	**10.6 ± 1.8**^**a**^	26.9	**175.3 ± 65.7**^**a,b**^	60.5	15.7 (*S*)
2. Tla Taghramt	**12.5 ± 0.8**^**a**^	10.8	**239.4 ± 31.6**^**a**^	21.3	19.3 (*S/CS*)
3. Ksar Sghir	2.6 ± 0.4^e,f^	27.1	15.3 ± 3.7^c^	39.5	10.1 (*S*)
(B) Dry subhumid climate					
4. Bni Harchin	5.7 ± 1.8^b,c^	52.0	**170.4 ± 80.4**^**a,b**^	76.1	18.5 (*S/CS*)
5. Dar Chaoui	4.0 ± 1.3^c,d,e,f^	52.6	29.5 ± 9.6^c^	52.7	11.2 (*S*)
6. Bni Arous	4.3 ± 1.2^b,c,d,e,f^	44.6	70.0 ± 39.7 ^c^	91.5	16.5 (*S/CS*)
7. Tnin Sidi Yamani	5.6 ± 0.4^b,c^	11.0	62.3 ± 8.4 ^c^	21.8	18.4 (*S/CS*)
8. Dar Akoubaa	6.7 ± 0.9^b^	22.7	**205.2 ± 69.9**^**a**^	55.0	13.9 (*S*)
9. Ouezzene	3.5 ± 0.5^c,d,e,f^	23.7	24.1 ± 7.6 ^c^	50.9	10.3 (*S*)
10. Mesmouda	2.9 ± 0.5^e,f^	25.4	23.0 ± 7.8 ^c^	54.4	11.7 (*S*)
(C) Semi-arid climate					
11. Ras Ejery	4.2 ± 0.7^c,d,e,f^	25.8	18.8 ± 5.6 ^c^	47.8	13.1 (*S*)
12. Bouqachmir	3.9 ± 1.2 ^c,d,e,f^	48.3	40.2 ± 11.8 ^c^	47.4	13.4 (*S*)
13. Moulay Bouazza	4.4 ± 0.6^b,c,d,e^	23.5	60.2 ± 7.9 ^c^	21.2	12.1 (*S*)
14. Sebt Ait-Rahou	4.4 ± 0.5 ^b,c,d,e,f^	20.1	73.1 ± 19.9 ^c^	44.0	14.9 (*S*)
15. Oulad Aissa	5.3 ± 0.5^b,c,d^	14.5	97.9 ± 14.1^b,c^	23.3	12.4 (*S*)
16. El Ksiba	3.8 ± 0.8^c,d,e,f^	35.0	37.0 ± 11.1 ^c^	48.6	9.7 (*S*)
17. Bin Ouidane	2.0 ± 0.2^f^	17.1	17.1 ± 4.4 ^c^	41.8	9.1 (*S*)
18. Asni	3.0 ± 0.7^d,e,f^	38.7	42.2 ± 14.5 ^c^	55.2	12.9 (*S*)
19. Ouirgane	4.2 ± 0.6^c,d,e,f^	24.2	63.0 ± 9.6 ^c^	24.7	13.8 (*S*)
Mean	4.9 ± 1.3	59.4	77.1 ± 37.5	108.3	
X *v σ* (*r*)	0.810***		0.453 ns		
ANOVA *F*_*19*,*190*_	30.3***		17.4***		

CV identifies coefficient of variation at the intra-population level expressed as a percentage. For CSR strategies, calculated following Pierce et al. [4] values for ruderal (R) were always 0%. In consequence only data for % competitive (C) are presented. A significant Pearson r correlation between mean (X) and standard deviation (σ) justifies the use of CV in comparisons of trait variability (see Materials and Methods).

Values with the same suffix are not statistically significantly different at *P* < 0.05 in Tukey HSD post hoc tests with groupings with highest trait values in bold and the lowest with a grey background. Functional’ and ‘mechanistic’ traits are identified as prefixes using the same notation as in Table 2. The level of significance is expressed as follow

*** *P* < 0.001; ns, not statistically significant.

### Variation in trait expression between and within populations

For each trait studied, statistically significant differences were detected between populations and for all but one of their CV values (Tables 3, 4, 5 and 6). The size of the variance ratio, *F*, differed considerably and traits with the highest value of *F* in Tables 3, 4, 5 and 6 also exhibited more of their percentage variance at the highest hierarchical level (i.e. between populations; Table 7). Thus, with the exception of CHL, ‘ecophysiological’ leaf traits, mostly classified as ‘mechanistic’, had a higher value for the variance ratio than those relating to ‘morphological size and shape’ and to ‘structural allocation’ (Tables 3, 4 and 5). They also contained 50 to 80 percent of the total variance ‘between populations’ (Table 4). At the opposite end of the spectrum were LDMC and SLA, two key ‘functional’ traits in the ‘worldwide leaf economics spectrum’ [5], and LL/LL_max_. These traits appeared inherently plastic, with 48 to 61 percent variance between leaves on the same tree. A majority of morphological traits (LA, LL, LL_max_, LL/LW) were intermediate with the percentage variance slightly higher at the ‘population’ level (42–48%). For the remainder, variance in trait expression was distributed relatively evenly between the three hierarchical levels.

**Table 7:** Estimated percentage variance across hierarchical levels (site:tree:leaf) patterns differently for contrasted groupings of leaf traits.

Trait	% of variance			
	Sites	Tree	Leaf	Residual
Ecophysiological				
^m^CHL^*l*^	17.0	24.6	**50.1**	8.3
^m^DS^*s*^	**74.0**	6.6	16.0	3.4
^m^LWC^*s*^	**79.9**	7.4	10.3	2.5
^m^SLWC^*s*^	**75.6**	7.3	14.0	3.1
^f,m^LT^*s*^	**50.6**	16.2	27.9	5.3
Morphological				
^f,m^LA^***t*- f,m**^	25.0	**43.4**	26.3	5.3
^f,m^LL^***t*- f,m**^	27.6	**47.8**	20.2	4.4
^f,m^LW^*l*^	25.6	33.6	34.4	6.4
^f,m^LL_max_^*t*^	23.7	**43.5**	27.3	5.4
^m^LL/LW^*t*^	25.8	**42.8**	26.2	5.3
^m^LL/LL_max_^*l*^	13.2	16.2	**61.0**	9.6
Structural				
^f^LWM^*t*^	30.6	33.5	30.2	5.8
^f^LDM^*t*^	29.3	36.5	28.6	5.6
^f^SLA^*l*^	19.5	24.2	**48.2**	8.1
^f^LDMC^*l*^	14.8	29.1	**48.0**	8.2

Here and in the remaining Tables, the italicized first letter added as a suffix to the trait identifies the hierarchical level with the maximum value for variance and additionally very high values (> 40%) are in bold. ‘Functional’ and ‘mechanistic’ traits are identified as prefixes using the same notation as in Table 2.

### Interrelationships between traits and the identification of syndromes of co-occurring traits

Correlation matrices for the traits and for their CV values are presented as Table 8 and S2 Table, respectively. Correlations between traits (Table 8) ranged in number (and percentage) from CHL (0, 0%) to, at the other extreme, DBH (9, 53%), with SLA (6, 35%) and LDMC (7, 41%). The mean number of correlations with other traits ± standard deviation could be roughly ordered as: ‘ecophysiological’ [‘mechanistic’] (4.0 ± 3.3, n = 5) < ‘morphological’ [‘intermediate’] (5.7 ± 2.5, n = 6) < ‘structural’ [‘functional’] (7.0 ± 0.8, n = 4) = ‘whole plant’ [‘functional’] (6.5 ± 3.5, n = 2). Fewer statistically significant relationships were detected in the correlation matrix for CV values and of these, a disproportionately high 40 percent related to ‘structural’ traits including SLA and LDMC (S2 Table).

**Table 8:** Correlation matrix for trait values (n = 18). Site 13 (Moulay Bouazza) with exceptionally high values for LWC, SLWC and LT has been excluded from this and subsequent analyses.

Traits	CHL	DS	LWC	SLWC	LT	LA	LW	LL	LL_max_	LL/LW	LL/LL_max_	LWM	LDM	SLA	LDMC	H
Ecophysiological																
DS	-0.102															
LWC	-0.056	**-0.580**														
SLWC	-0.156	**-0.573**	**0.628**													
LT	0.262	-0.184	-0.013	0.125												
Morphological																
LA	0.066	-0.282	**0.759**	-0.019	-0.170											
LW	0.006	-0.383	**0.746**	0.157	-0.414	**0.853**										
LL	0.189	-0.164	**0.589**	-0.119	0.263	**0.834**	0.447									
LL_max_	-0.008	-0.341	**0.737**	0.164	0.201	**0.803**	**0.555**	**0.849**								
LL/LW	0.151	0.169	-0.004	-0.259	**0.620**	0.159	-0.360	**0.669**	0.421							
LL/LL_max_	0.321	0.272	-0.350	-0.387	0.168	-0.161	-0.331	0.049	-0.458	0.319						
Structural																
LWM	0.089	**-0.573**	**0.944**	0.464	0.215	**0.815**	**0.681**	**0.774**	**0.866**	0.239	-0.286					
LDM	0.287	-0.451	**0.643**	0.117	**0.530**	**0.704**	0.409	**0.881**	**0.870**	**0.568**	-0.119	**0.859**				
SLA	-0.271	0.233	0.138	-0.095	**-0.933**	0.287	**0.503**	-0.127	-0.064	**-0.537**	-0.183	-0.086	-0.425			
LDMC	0.289	0.096	-0.454	**-0.543**	**0.694**	-0.164	**-0.474**	0.244	0.008	**0.642**	0.399	-0.157	0.341	**-0.766**		
Whole Plant																
H	-0.166	0.031	0.447	0.141	**-0.561**	0.467	**0.610**	0.130	0.339	-0.361	-0.419	0.316	0.039	**0.639**	**-0.626**	
DBH	-0.073	-0.145	**0.625**	0.340	-0.333	**0.521**	**0.606**	0.296	**0.543**	-0.179	-**0.512**	**0.534**	0.271	**0.503**	**-0.618**	**0.879**

Here and in Table 9, values relate to Pearson r and statistically significant values at P < 0.05 are in bold.

With regard to estimates of CSR strategy, which is described in the Introduction, populations were classified variously as stress-tolerant competitors (SC) and as intermediate between this strategy and stress-tolerators (S/SC), with percentage values ranging from 9–19 for the competitive (C) dimension, 81–91 for stress-tolerance (S) and consistently zero for ruderality (R; Table 6). In the PCA analyses of traits (Table 9) axis 1 identified size and was positively correlated with plant dimensions (DBH, H) and a plethora of size-related leaf traits (LA, LL, LL_max_, LW, LWC, LWM, LDM). Axis 2 included aspects of the ‘worldwide leaf economics spectrum’ [5,6] with LT and LDMC (both positively) and SLA (negatively) impacting upon the expression of the axis. High values on axis 3 was identified by small leaves (LL^–^), a high water content (SLWC^+^), high leaf construction costs (SLA^–^) and low stomatal density (DS^–^). We need to investigate whether trait expression in site 13, a functional outlier, excluded from the analyses because of its high values for LT (value 125% that of the next highest site average; Table 3), LWC (>300%) and SLWC (>400%), is influenced by similar factors to those defining this axis. The PCA analysis for traits plus CV values, included for completeness as S3 Table, generated broadly similar results.

**Table 9:** The traits that define ‘syndromes’: correlations between traits and the three PCA axes identified.

Trait	PCA 1	PCA 2	PCA 3
Ecophysiological			
CHL^***l***^	-0.017	0.380	-0.119
DS^***s***^	**-0.488**	-0.161	**-0.631**
LWC^***s***^	**0.936**	0.020	0.231
SLWC^***s***^	0.396	-0.160	**0.829**
LT^***s***^	-0.104	**0.864**	0.351
Morphological			
LA^***t***^	**0.877**	0.119	-0.387
LW^***t***^	**0.832**	-0.281	-0.167
LL^***l***^	**0.688**	**0.579**	**-0.403**
LL_max_^***t***^	**0.862**	0.364	-0.106
LL/LW^***t***^	0.026	**0.825**	-0.289
LL/LL_max_^***l***^	-0.458	0.328	-0.348
Structural			
LWM^***t***^	**0.928**	0.312	0.135
LDM^***t***^	**0.703**	**0.700**	-0.026
SLA^***l***^	0.240	**-0.840**	**-0.414**
LDMC^***l***^	-0.393	**0.831**	-0.180
Whole Plant			
H	**0.598**	**-0.583**	-0.244
DBH	**0.753**	-0.393	-0.075
Eigenvalues	6.66	4.76	2.11
Variance (%)	39.19	28.05	12.46

Eigenvalues and percentage variance explained by each axis are included below the list of traits (Table 9).

### Correlates with climate

Each site has its own characteristic climatic regime (Table 1). As a result, for a canopy dominant such as oleaster, traits that pattern most strongly with site are also more likely to pattern with climate. Consistent with this, the traits that varied most between sites (with the suffix ^*s*^) had the greatest percentage of statistically significant correlations with climatic indices (45%; Table 10A). These traits were classified as ecophysiological/’mechanistic’. In contrast, for traits where more variance was expressed within the same population (morphological/‘intermediate’, ^*t*^) or between leaves on the same tree (leaf/‘functional’, ^*l*^), the percentage of statistically significant correlations was lower (25% and 6% respectively; Table 10A).

**Table 10:** Correlations between climatic indices and (A) traits and (B) coefficient of variation (CV).

	(A) Trait and syndrome values (Pearson *r*)					(B) CV (Spearman *r*)				
	MAT	MAP	MTCM	AI	Altitude	MAT	MAP	MTCM	AI	Altitude
**TRAITS**										
Ecophysiological: *n* = 18						*n* = 18				
CHL^*l*^	-0.058	-0.116	-0.007	-0.112	0.031	0.401	0.346	**0.513**	**0.489**	**-0.575**
DS^*s*^	**-0.549**	**-0.534**	**-0.569**	**-0.495**	**0.586**	0.337	-0.013	0.137	-0.019	-0.321
LWC^*s*^	0.130	0.346	0.387	0.385	-0.432	0.193	0.053	0.108	-0.126	-0.331
SLWC^*s*^	0.463	**0.500**	**0.581**	**0.541**	**-0.595**	0.211	0.183	0.240	0.148	-0.379
LT^*s*^	0.017	0.031	-0.149	-0.087	0.117	-0.190	-0.148	0.143	0.101	0.015
Morphological: *n* = 18										
LA^*t*^	-0.230	0.003	0.019	0.036	-0.053	0.003	-0.127	0.049	-0.077	-0.003
LL^*t*^	-0.049	0.282	0.367	0.354	-0.333	0.177	-0.049	0.176	0.135	-0.069
LW^*l*^	-0.311	-0.225	-0.305	-0.260	0.221	-0.120	-0.106	-0.092	-0.170	0.013
LL_max_^*t*^	0.033	0.069	0.068	0.079	-0.092	0.355	**0.503**	0.453	0.438	-0.434
LL/LW^*t*^	-0.306	**-0.480**	**-0.647**	**-0.581**	**0.535**	0.299	-0.228	0.022	-0.113	0.036
LL/LL_max_^*l*^	-0.463	-0.298	**-0.483**	-0.391	0.373	**0.485**	**0.804**	**0.711**	**0.701**	**-0.672**
Structural: *n* = 18										
LWM^*t*^	0.055	0.263	0.244	0.266	-0.293	0.055	-0.015	0.024	-0.184	-0.139
LDM^*t*^	-0.047	0.093	-0.018	0.036	-0.033	0.202	-0.022	0.265	0.129	-0.112
SLA^*l*^	-0.065	-0.081	0.152	0.070	-0.104	0.277	0.298	0.152	0.126	-0.276
LDMC^*l*^	-0.206	-0.226	**-0.477**	-0.382	0.428	**0.480**	0.290	**0.476**	0.290	**-0.620**
Whole plant: *n* = 18										
H	-0.103	0.350	0.358	**0.492**	-0.274	-0.013	0.067	0.199	0.178	-0.156
DBH	0.007	0.289	0.350	0.420	-0.248	0.114	0.321	0.168	0.239	-0.216
**‘SYNDROMES’**										
C strategy										
C%	0.003	0.243	0.372	0.351	-0.380					
PCA axis1	-0.033	0.158	0.292	0.304	-0.294	0.349	0.240	0.432	0.250	**-0.503**
axis 2	-0.267	-0.240	**-0.521**	**-0.483**	0.401	-0.114	-0.018	-0.170	-0.127	0.036
axis 3	**0.687**	**0.552**	**0.645**	**0.523**	**-0.647**	-0.279	-0.236	-0.075	-0.128	0.053

As in Table 1, climatic indices are abbreviated as follows: mean annual temperature, MAT, °C; mean temperature of coldest month, MTCM, °C; mean annual precipitation, MAP, mm; aridity index, AI (MAP/PET where PET identifies potential evapotranspiration, mm).

The traits most consistently correlated with climate were DS and leaf shape (LL/LW), negatively, and SLWC, positively correlated with mean annual precipitation (MAP), minimum temperature of the coldest month (MTCM), global aridity index (AI) and Altitude (Table 10A). The only trait significantly correlated with mean annual temperature (MAT) was DS (Table 10A). Importantly, SLA, a ‘flagship’ trait in both the ‘leaf economics spectrum’ and, more generally, in trait-based plant ecology, was one of the traits not correlated directly with any of the climatic variables.

Of the syndromes of traits, PCA axis 3, which explained only 12% of the variation in the dataset (Table 9), was correlated with all five climatic-related variables (Table 10A). In addition, PCA axis 2 patterned with MTCM and AI. However, PCA axis 1 and CSR strategies showed no significant correlations. Moreover, the additional inclusion of CV in PCA analyses axes reduced rather than enhanced the extent of correlation with climatic variables (S3 Table) and in separate analyses of CV values only the traits LL/LL_max_, LDMC and CHL were frequently correlated with climate (Table 10B).

## Discussion

### The traits and trait syndromes that pattern with climate± and those that don’t

A basic premise in much of trait-based ecology is that taxa may be grouped using physiological and morphological traits into functional types, with taxa in the same functional group displaying similar responses to the environment [119,120]. Consistent with this, major PCA analyses of the world flora have routinely identified two key dimensions of functional specialization, the ‘worldwide leaf economics spectrum’ and size [12,13]. Here, for populations within a single species, oleaster, we confirm these generalities. Axes 1 and 2 in our PCA analysis are analogous with the same two axes recognized in global studies (Tables 3–10). However, these axes did not consistently pattern with climatic variables. Instead, a further ‘climatic’ PCA axis 3, was identified defined primarily in terms of ecophysiological/’mechanistic’ leaf traits (Table 10A). Equally, a majority of the statistically significant correlations between individual traits and climate included ecophysiological/’mechanistic’ leaf traits rather than the more commonly used structural/‘functional’ ones (Table 10A). However, a precise interpretation in terms of ecological processes is not yet feasible. Both the origin and the generality of variability in trait expression are in need of further study. Oleaster is slow-growing and long-lived [56,59] and will experience variations in climate during its lifetime. Moreover, it exhibits high levels of genetic heterozygosity [54,121–127]. Variability in trait expression will have been affected by genotype, phenotypic plasticity [16,128] and potentially differences in phenology and in the land-use and climatic history of the study sites. Furthermore, a different choice of species may have led to a very different set of results and conclusions: ‘functional’ traits are predicted to pattern more strongly with climate for fast-growing and short-lived species (Fig 1). Nevertheless, these equivocations do not alter the basic message of this study. ‘Mechanistic’ traits must be routinely included in climate-related studies.

### A preliminary assessment of the ‘climatic’ PCA 3 axis in oleaster

Oleaster is a thermophilic species with limited resistance to both cold and extreme aridity [55]. Moreover, aridity is generally regarded as the key climatic factor determining species composition in Mediterranean vegetation. The ‘climatic’ PCA axis 3 was correlated positively with MAT, MAP, MTCM and AI and negatively with altitude (Table 10A). Thus, high values of PCA axis 3 equated to high mean annual temperature, high minimum temperature of the coldest month, high mean annual precipitation, high aridity and low altitude. In terms of the traits that define PCA axis 3 (Table 9), these climatic extremes were associated with low stomatal density (DS^–^) low leaf water content (SLWC^–^), small leaves (LL^–^) and high leaf construction costs (SLA^+^). The trends for DS were similar to those recorded elsewhere in relation to climate [129,130]. Similarly, elongate leaves with a potentially thinner boundary layer [99,131,132] tended to be associated with harsher climates. PCA axis 3 may identify a water-use/succulence dimension. Moreover, site 13, excluded for its extremely high values for LT, LWC and SLWC (Table 3), may perhaps represent an extremely ‘succulent’ outlying population.

Succulence is an ecologically important functional mechanism defined as the ‘storage of utilizable water in living tissues in one or several plant parts in such a way as to allow the plant to be temporarily independent from external water supply but to retain at least some physiological activity’ [133]. However, structural and physiological relationships involving succulence are complex [133–136]. As a result, whether PCA axis 3 identifies ‘succulence’, and provides a temporary drought avoidance mechanism, still requires experimental verification. Nevertheless, whatever the exact functional origins of climate-related PCA axis 3, its recognition was, importantly, only made possible through the inclusion of several traits not customarily included in trait-based ecological studies.

### The way forward–an olive branch in the ‘functional’ versus ‘mechanistic’ debate

Our results, and most of the relationships included in our literature review, point to ‘mechanistic’ traits being diagnostically superior to ‘functional’ ones in climate studies. Nevertheless, we believe it counterproductive to focus entirely on this ‘mechanistic’ superiority. To re-iterate arguments from the Introduction, ‘mechanistic’ studies are time-consuming with small datasets produced and species chosen primarily for ecological relevance. Without a level of co-ordination not currently in place between related ‘mechanistic’ investigations, precision of measurement will be offset by few shared species and no integration of findings (i.e. little generality). Trait based ecology, and ‘functional’ traits, can potentially provide this missing generality by producing large ecologically-balanced datasets. However, as in this study, results may require mechanistic clarification. Importantly, as currently implemented, trait-based ecology is methodologically flawed. Specialization routinely occurs at the cellular/biochemical level but may additionally be identified at a higher organizational scale by measuring organs or whole plants. So far, few suitable methodologies are available for the large-scale measurement of traits that can only be identified at the lowest, most fundamental cellular/biochemical level. As a result, the size of plants and their parts, plus SLA and LDMC, which can be measured using whole leaves, stand imprecisely at the centre of meta-analyses in trait-based ecology [12,13]. In contrast, some important ecological specializations expressed primarily at the cellular/biochemical level (e.g. aluminium tolerance in acidic soils and restriction by climate [47,137]) remain outside the scope of these meta-analyses. Moreover, this study only partly redresses this ‘climatic imbalance’. Many addition simply-measured ‘mechanistic’ traits will be required to routinely add a comprehensive climatic dimension to trait-based studies.

Volaire [48] identified ‘functional’ traits as dealing with ecological strategies relating to multi-environmental factors and studied implicitly over long timescales. This is true but, equally, it is the product of methodological constraints rather than an ideological choice. Both the ‘mechanistic’ and ‘functional’ trait approaches have strengths, and weaknesses, but similar goals. In our search to understand and quantify impacts of changing climate on global vegetation composition and ecosystem function, the challenge will be to combine the strengths of both approaches–and to use both types of traits. ‘Mechanistic’ and ‘functional’ traits both contribute to our proposed plant-climate model (Fig 1) and each may be expected to add more generally to our understanding of climate-related processes.

## Supporting information

S1 Table Percentage coefficient of variation (CV) for leaf functional traits including minimum (CV_min_) and maximum values CV_max_). (DOCX)

S2 Table Correlation matrix for CV values of traits (n = 18) with statistically significant relationships in bold. (DOCX)

S3 Table The combinations of traits and CVs that define ‘syndromes’. (DOCX)
