# Supplementary material for: Trait-based plant ecology a flawed tool in climate studies? The leaf traits of wild olive that pattern with climate are not those routinely measured
Source: PLoS One. 2019 Jul 17;14(7):e0219908. doi: 10.1371/journal.pone.0219908 (PMC6636763; doi:10.1371/journal.pone.0219908)
Supplement: S1 Table — (DOCX) [file pone.0219908.s001.docx]

**S1 Table.** **Percentage coefficient of variation (CV) for leaf functional traits including minimum (CV_min_) and maximum values CV_max_).**

| Trait | CV | CV_min_ | CV_max_ | CM |
| --- | --- | --- | --- | --- |
| Ecophysiological | | | | |
| CHL^m^ | 15.77 | 6.11 | 30.05 | **0.662** |
| DS^m^ | 23.56 | 7.28 | 26.27 | **0.566** |
| LWC^m^ | **85.42** | 12.37 | 66.47 | **0.686** |
| SLWC^m^ | **105.51** | 25.31 | 92.05 | **0.569** |
| LT^f,m^ | 20.93 | 8.25 | 27.8 | **0.542** |
| Morphological | | | | |
| LA^f,m^ | 41.71 | 28.46 | 42.13 | 0.194 |
| LL^f,m^ | 26.60 | 16.09 | 31.22 | 0.320 |
| LW^f,m^ | 23.23 | 13.98 | 27.27 | 0.322 |
| LL_max_^f,m^ | 32.26 | 12.24 | 40.84 | 0.539 |
| LL/LW^m^ | 26.14 | 14.72 | 29.31 | 0.331 |
| LL/ LL_max_^m^ | 22.12 | 8.72 | 39.08 | **0.635** |
| Structural | | | | |
| LWM^f^ | 44.06 | 27.7 | 45.16 | 0.240 |
| LDM^f^ | 49.92 | 29.01 | 53.34 | 0.295 |
| SLA^f^ | 40.94 | 24.32 | 63.55 | 0.446 |
| LDMC^f^ | 29.84 | 18.89 | 38.7 | 0.344 |

Canberra metric values (CM) calculated as CM = [(CV_max_ - CV_min_)/(CV_max_ + CV_min_)]. High and low values identified in bold and by grey background respectively. ‘Functional’ and ‘mechanistic’ traits are identified as prefixes using the same notation as in Table 2.
