# Supplementary material for: Trait-based plant ecology a flawed tool in climate studies? The leaf traits of wild olive that pattern with climate are not those routinely measured
Source: PLoS One. 2019 Jul 17;14(7):e0219908. doi: 10.1371/journal.pone.0219908 (PMC6636763; doi:10.1371/journal.pone.0219908)
Supplement: S2 Table — (DOCX) [file pone.0219908.s002.docx]

**S2 Table.** **Correlation matrix for CV values of traits (n =18) with statistically significant relationships in bold.**

| Variables | CHL | DS | LWC | SLWC | LT | LA | LW | LL | LL_max_ | LL/LW | LL/LL_max_ | LWM | LDM | SLA | LDMC | H |
| --- | --- | --- | --- | --- | --- | --- | --- | --- | --- | --- | --- | --- | --- | --- | --- | --- |
| Ecophysiological | | | | | | | | | | | | | | | | |
| CHL^m^ |  |  |  |  |  |  |  |  |  |  |  |  |  |  |  |  |
| DS^m^ | 0.137 |  |  |  |  |  |  |  |  |  |  |  |  |  |  |  |
| LWC^m^ | 0.010 | 0.030 |  |  |  |  |  |  |  |  |  |  |  |  |  |  |
| SLWC^m^ | -0.059 | 0.051 | **0.792** |  |  |  |  |  |  |  |  |  |  |  |  |  |
| LT^f,m^ | 0.084 | 0.363 | -0.400 | -0.395 |  |  |  |  |  |  |  |  |  |  |  |  |
| Morphological | | | | | | | | | | | | | | | | |
| LA^f,m^ | -0.045 | 0.344 | 0.379 | 0.245 | 0.092 |  |  |  |  |  |  |  |  |  |  |  |
| LW^f,m^ | 0.104 | 0.194 | 0.394 | 0.171 | 0.097 | **0.680** |  |  |  |  |  |  |  |  |  |  |
| LL^f,m^ | 0.094 | 0.282 | 0.043 | 0.087 | -0.151 | **0.512** | -0.048 |  |  |  |  |  |  |  |  |  |
| LL_max_^f,m^ | -0.015 | 0.168 | 0.366 | 0.116 | 0.027 | **0.566** | **0.714** | -0.044 |  |  |  |  |  |  |  |  |
| LL/LW^m^ | 0.050 | -0.050 | -0.088 | -0.246 | -0.196 | 0.040 | 0.438 | -0.248 | 0.063 |  |  |  |  |  |  |  |
| LL/LL_max_^m^ | -0.074 | 0.124 | -0.061 | -0.169 | -0.061 | 0.139 | 0.050 | -0.098 | **0.615** | 0.005 |  |  |  |  |  |  |
| Structural | | | | | | | | | | | | | | | | |
| LWM^f^ | -0.089 | 0.166 | **0.760** | 0.365 | -0.045 | **0.691** | **0.603** | 0.131 | **0.608** | 0.037 | 0.153 |  |  |  |  |  |
| LDM^f^ | 0.095 | **0.479** | -0.259 | -0.287 | **0.570** | **0.569** | 0.406 | 0.308 | 0.377 | 0.104 | 0.247 | 0.289 |  |  |  |  |
| SLA^f^ | -0.030 | 0.414 | 0.194 | 0.006 | 0.132 | 0.427 | 0.380 | 0.267 | **0.608** | -0.027 | 0.449 | **0.473** | **0.536** |  |  |  |
| LDMC^f^ | 0.367 | **0.586** | 0.398 | 0.154 | 0.133 | 0.377 | **0.482** | 0.136 | **0.551** | 0.146 | 0.311 | **0.540** | 0.402 | **0.522** |  |  |
| Whole Plant | | | | | | | | | | | | | | | | |
| H^f^ | 0.225 | 0.403 | 0.440 | **0.532** | -0.023 | 0.246 | 0.377 | -0.105 | 0.237 | -0.003 | -0.071 | 0.225 | -0.050 | -0.048 | 0.172 |  |
| DBH^f^ | 0.041 | 0.381 | 0.346 | 0.215 | -0.214 | 0.388 | 0.449 | 0.068 | **0.478** | 0.154 | 0.265 | 0.382 | 0.018 | 0.121 | 0.315 | **0.717** |

Site 13 (Moulay Bouazza) with exceptionally high values for LWC, SLWC and LT has been excluded from this and subsequent analyses. Here and in Table S2, values relate to Spearman r and statistically significant values at P < 0.05 are in bold. ‘Functional’ and ‘mechanistic’ traits are identified as prefixes using the same notation as in Table 2.
