# Supplementary material for: Trait-based plant ecology a flawed tool in climate studies? The leaf traits of wild olive that pattern with climate are not those routinely measured
Source: PLoS One. 2019 Jul 17;14(7):e0219908. doi: 10.1371/journal.pone.0219908 (PMC6636763; doi:10.1371/journal.pone.0219908)
Supplement: S3 Table — (DOCX) [file pone.0219908.s003.docx]

**S3 Table.** **The combinations of traits and CVs that define ‘syndromes’.**

| Trait | PCA 1 | PCA 2 | PCA 3 |
| --- | --- | --- | --- |
| Ecophysiological |  |  |  |
| CHL^m^ | -0.037 | 0.303 | 0.214 |
| *CHL_cv* | 0.209 | -0.269 | -0.175 |
| DS^m^ | **-0.586** | -0.225 | 0.180 |
| *DS_cv* | **0.559** | 0.071 | -0.268 |
| LWC^m^ | **0.949** | 0.013 | 0.033 |
| *LWC_cv* | 0.042 | **0.681** | -0.297 |
| SLWC^m^ | **0.563** | 0.195 | -0.631 |
| *SLWC_cv* | 0.047 | **0.579** | -0.067 |
| LT^f,m^ | -0.034 | **0.848** | 0.222 |
| *LT_cv* | 0.290 | **-0.620** | -0.106 |
| Morphological |  |  |  |
| LA^f,m^ | **0.756** | -0.180 | 0.536 |
| *LA_cv* | **0.677** | 0.377 | -0.191 |
| LW^f,m^ | **0.716** | **-0.481** | 0.227 |
| *LW_cv* | **0.677** | 0.226 | 0.527 |
| LL^f,m^ | **0.610** | 0.262 | 0.695 |
| *LL_cv* | 0.351 | 0.296 | -0.577 |
| LL_max_^f,m^ | **0.782** | 0.056 | 0.526 |
| *Ll_max__cv* | **0.490** | 0.291 | -0.582 |
| LL/LW^m^ | 0.040 | **0.661** | **0.541** |
| *LL/LW_cv* | -0.219 | 0.142 | -0.379 |
| LL/LL_max_^m^ | -0.387 | **0.435** | 0.078 |
| Structural |  |  |  |
| LWM^f^ | **0.915** | 0.180 | 0.276 |
| *LWM_cv* | 0.328 | **0.475** | -0.430 |
| LDM^f^ | **0.655** | 0.414 | **0.582** |
| *LDM_cv* | **0.702** | -0.248 | -0.156 |
| SLA^f^ | 0.184 | **-0.834** | -0.173 |
| *SLA_cv* | **0.567** | 0.249 | -0.145 |
| LDMC^f^ | **-0.429** | **0.598** | **0.535** |
| *LDMC_cv* | **0.608** | 0.075 | **-0.546** |
| Whole Plant |  |  |  |
| H^f^ | **0.465** | **-0.651** | 0.033 |
| *H_cv* | 0.050 | **0.452** | **-0.433** |
| DBH^f^ | **0.704** | **-0.450** | -0.009 |
| *DBH_cv* | 0.219 | **0.481** | **-0.554** |
| Eigenvalues | 9.17 | 6.18 | 5.17 |
| Variance (%) | 26.98 | 18.18 | 15.21 |

Correlations between traits and PCA axes. Eigenvalues and percentage variance explained by each axis are also included. ‘Functional’ and ‘mechanistic’ traits are identified as prefixes using the same notation as in Table 2.
